# Protocol to establish endogenous knockins in Jurkat T-ALL cells using CRISPR-Cas9-mediated homology-directed repair

**DOI:** 10.1016/j.xpro.2026.104817

**Published:** 2026-09-11

**Authors:** Joana R. Costa, Yang Li, Sara Ahrabi, Zhaodong Li, Tanya Rapoz-D’Silva, Mateja E. Krulik, Sara Hyseni, George Morrow, Yanping Guo, A. Thomas Look, Marc R. Mansour

**Affiliations:** 1 Department of Haematology, UCL Cancer Institute, University College London, London, UK; 2 Department of Pediatric Oncology, Dana-Farber Cancer Institute, Harvard Medical School, Boston, MA, USA; 3 Faculty of Biology, University of Wurzburg, Wurzburg, Germany; 4 Flow Cytometry Translational Technology Platform, University College London, London, UK; 5 Division of Pediatric Hematology-Oncology, Boston Children’s Hospital, Boston, MA, USA; 6 Developmental Biology and Cancer, UCL Great Ormond Street Institute of Child Health, London, UK; 7 University College London Hospitals NHS Foundation Trust, London, UK

**Keywords:** Cell Biology, Cell culture, Cell-based Assays, Flow Cytometry, Cancer, Molecular Biology, CRISPR

## Abstract

Jurkat cells are a versatile cell-line model utilized across multiple areas of biology. Here, we present a protocol for inserting DNA sequences (knockins) at an endogenous locus using CRISPR-Cas9 technology via homology-directed repair (HDR). We describe steps for designing, constructing, and validating endogenous knockins in T-ALL Jurkat cells. This protocol has potential application in gene regulatory studies, cancer biology, HIV research, and T-cell signaling, among others.

For complete details on the use and execution of this protocol, please refer to Costa JR et al.^1^

## Highlights

• Instructions for creating a GFP-oncogene fused protein using CRISPR-Cas9-HDR
• Steps for introducing precise edits within non-coding regulatory sequences
• Guidance on reagent design, construction, and delivery

## Abstract (editor highlights)

Publisher’s note: Undertaking any experimental protocol requires adherence to local institutional guidelines for laboratory safety and ethics.

## Graphical abstract

**Figure:**
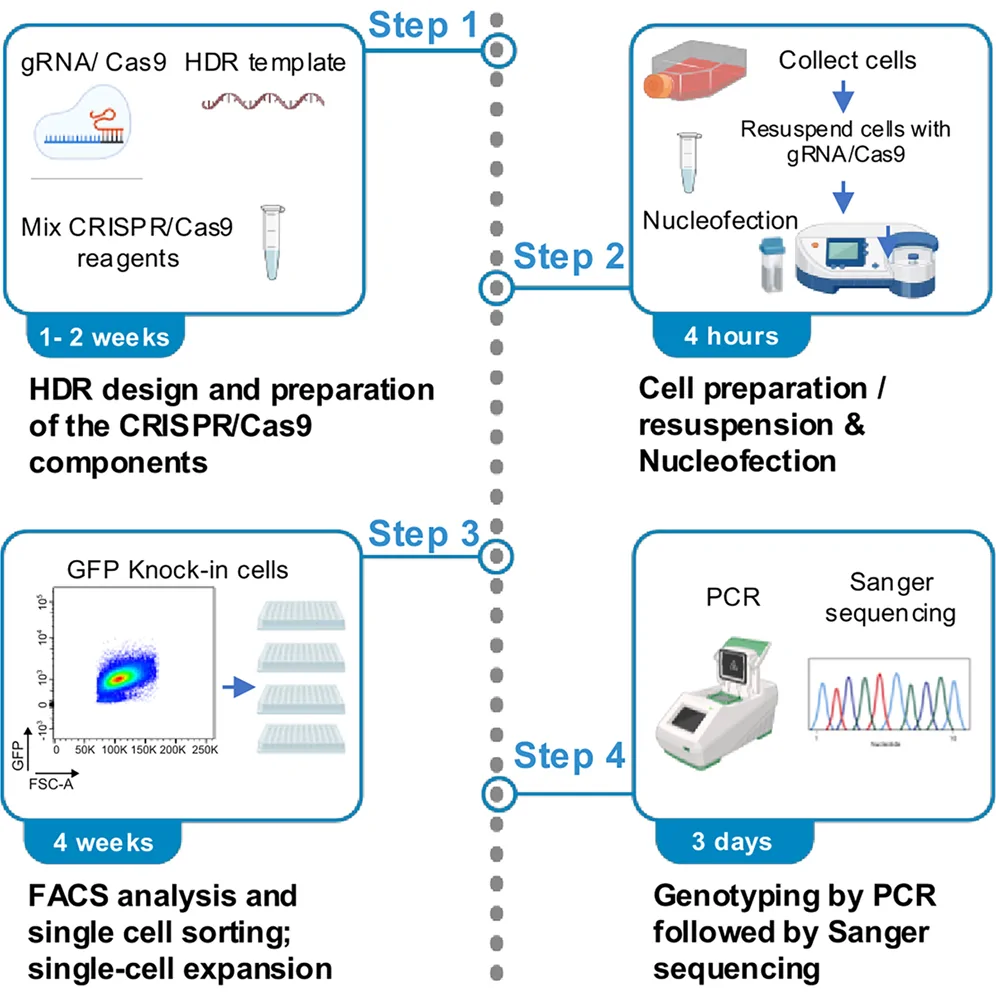

## Before you begin

Jurkat cells were first established in the 1970s, originating from a 14 year old boy with T-cell acute lymphoblastic leukemia (T-ALL). They have proven to be a faithful model of T-ALL, harboring many of the mutations found in the disease, including the prototypic gain-of-function enhancer mutation at the *TAL1* oncogene. Due to their versatility, they have been widely co-opted in diverse areas of research, such as those involving T-cell receptor signaling, gene regulation, HIV infection, cancer biology, apoptosis and cytokine studies.

In recent years, the emergence of CRISPR-Cas9 technology has resulted in major advances in the field of genome editing, particularly in modifying genes and regulatory sequences at their endogenous loci, redefining our understanding of gene regulation at the native locus.

The combined strategy of CRISPR-Cas9 with improved sgRNA libraries^2^ and analysis pipelines^3^ has changed our abilities to investigate gene regulation through genetic screenings.

The CRISPR-Cas9 technology enables the insertion of large or short DNA sequences by using cellular DNA repair mechanisms such as Homology-directed repair (HDR).^4^ HDR is used to insert or modify large DNA sequences, such as fluorescent protein tags.

Attaching a fluorescent protein tag to a gene is a common approach for studying gene expression. Prior to the emergence of the CRISPR-Cas9 technology, this mainly relied on the exogenous expression of the reporter fused to the recombinant protein of interest, which frequently resulted in artifacts and did not adequately reflect endogenous gene regulation.

Here, we present a detailed protocol for creating a reporter Jurkat cell line in which a large knock-in comprising a GFP fluorescent tag is placed in-frame with an endogenous gene, utilizing the *TAL1* oncogene as an example. We have used a strategy where the HDR template harbors a DsRed marker to monitor transfection efficiency and to enable exclusion of cells where recombination has taken place in off-target loci. Following validation, the GFP-TAL1 cell line was used to perform a genetic screen to identify key regulators of the oncogene.^1^

We then investigated the functionality of the key regulators of the *TAL1* oncogene by precisely disrupting their recognition sites at the DNA regulatory sequence using CRISPR-Cas9-HDR through a single-stranded DNA oligonucleotide (ssODN). Accordingly, we used this technology to change the sequence of three GATA binding sites by one base pair. In addition, we employed CRISPR-Cas9-HDR to remove a 12-bp binding site sequence recognized by the transcription factor (TF) MYB.

This protocol can be adapted for performing endogenous knock-ins of other gene loci thus addressing fundamental questions related to gene regulation in cancer malignancies.

### Innovation

Studying gene regulation at the native genomic locus is essential for understanding gene function in a physiological context. Performing high efficiency CRISPR-Cas9-homology directed repair (HDR) has proven challenging, particularly in the non-coding regions of suspension cell lines.

Here, we present an optimized and detailed protocol for generating CRISPR-Cas9- HDR endogenous knock-in of a large insert tag (∼700 bp), enabling the investigation of oncogene regulation at its native locus. In addition, we provide an optimized approach for introducing precise CRISPR-Cas9-HDR edits, including single base-pair substitutions, within the regulatory region of the tagged oncogene.

Together, these strategies provide a robust, highly efficient and reproducible multi-site methodology to studying oncogene regulation in the native locus. The protocol described here is adaptable to other oncogenes of interest and can be applied to investigate regulatory mechanisms across diverse cellular models.

## Key resources table

**Table:** 

REAGENT or RESOURCE	SOURCE	IDENTIFIER
**Bacterial and virus strains**		

One shot Stbl3 chemically competent E.coli	Invitrogen	Cat# C737303

**Chemicals, peptides, and recombinant proteins**		

Recombinant Cas9	IDT	Cat# 1081061
tracrRNA-ATTO550	IDT	Cat# 1075928
Ingenio Electroporation Solution	Mirus	Cat# MIR50111
Phusion High-Fidelity DNA polymerase	NEB	Cat# M0530S
Hoechst 33258	Invitrogen	Cat# H3569

**Critical commercial assays**		

Miniprep kit	Qiagen	Cat# 27104
Midiprep kit	Qiagen	Cat# 12943
DNeasy Blood and Tissue Kit	Qiagen	Cat# 69504
QuickExtract DNA Extraction Solution	LGC, Biosearch Technologies	Cat# QE0905T

**Experimental models: Cell lines**		

Jurkat MSCV-TAL1	Mansour MR et al.^5^	N/A
Jurkat GFP-TAL1	This paper	N/A
Jurkat GFP-TAL1 GATA3 mutants	This paper	N/A
Jurkat GFP-TAL1 MuTE repaired	This paper	N/A

**Oligonucleotides**		

sgRNA sequence directed at TAL1 5′-CTGCCCACAGGATGACCGAGCGG-3′	IDT	N/A
sgRNA TAL1_sense 5′-CACCGCTGCCCACAGGATGACCGAG sgRNA TAL1_antisense 5′-AAACCTCGGTCATCCTGTGGGCAGC	IDT	N/A
sgRNA AAVS1: 5′- AGCGGCTCCAATTCGGAAGT – 3′	IDT	N/A
sgRNA MYB: 5’ – ACCAGGCACACAAGAGACTG – 3′	IDT	N/A

**Recombinant DNA**		

pUC57-kan	GenScript	
pX330-U6-Chimeric_BB-CBh-hSpCas9	Addgene	Cat# 42230

**Software and algorithms**		

BioRender		RRID:SCR_018361 https://www.biorender.com/
FlowJo	FlowJo Software	v10.9.0, RRID:SCR_008520 https://www.flowjo.com/
Benchling		RRID:SCR_013955https://www.benchling.com/

**Other**		

Amaxa Nucleofector II	Lonza Bioscience	RRID:SCR_022262
C-1000 Thermal Cycler	Bio-Rad	RRID:SCR_019688
BD LSR Fortessa Cell Analyzer	BD Biosciences	
BD FACSAria Fusion Flow Cytometer	BD Biosciences	

## Materials and equipment

**Table:** Phosphorylation and annealing of the crRNA oligonucleotides

Reagent	Final concentration	Amount
sgRNA oligo sense (100 μM)	10 μM	1 μl
sgRNA oligo antisense (100 μM)	10 μM	1 μl
10x T4 ligation buffer (NEB)	1x	1 μl
T4 PNK (NEB)	-	0.5 μl
H2O	-	Up to 10 μl

***Note:*** Phosphorylated and annealed crRNA oligonucleotides can be stored at −20^o^C for up to 24 months.

**Table:** Ligation of the sgRNA duplex into the vector

Reagent	Amount
Linearized vector (50ng/ul)	1 μl
Diluted crRNA duplex, from step 11	1 μl
10x T4 ligation buffer (NEB)	1 μl
T4 Ligase	0.5 μl
H2O	6.5 μl

• Incubate the ligation reaction for 2 h at 20^o^C −25^o^C. Heat-inactivate by incubating at 65^o^C for 15 min.

***Note:*** We recommend also setting up a no insert ligation control reaction.

**Table:** PCR reaction master mix

Reagent	Final concentration	Volume
gDNA	-	1μl
Primer F (10μM)	1μM	0.2μl
Primer R (10μM)	1μM	0.2μl
Phusion High-Fidelity DNA polymerase 2x	1x	12.5μl
H2O	-	Up to 25μl

• Prepare the PCR reaction on ice.

**CRITICAL:** Include a negative reaction with no added genomic DNA to ensure PCR reagents are not contaminated

**Table:** PCR cycling conditions

PCR step	Temperature	Duration	Cycles
Initial Denaturation	98°C	30 seconds	1
Denaturation	98°C	10 seconds	27 to 32
Annealing	Primer pair dependent	10–30 seconds	
Extension	72°C	15–30 seconds/Kb	
Final Extension	72°C	10 min	1
Hold	4°C-10°C	-	-

## Step-by-step method details

### Designing the donor plasmid template utilized for CRISPR-Cas9-HDR GFP knockin

**Timing: 1 day**

This section describes how to design the Homology Directed Repair (HDR) plasmid donor template used for the CRISPR-Cas9 knock-in of the GFP tag. The HDR template harbors a GFP sequence that is flanked by two-homology arms. The homology arms are sequences homologous to the gene of interest (e.g., *TAL1*) flanking the cut site.

We provide a strategy where the HDR template harbors a DsRed marker to monitor transfection efficiency and to enable exclusion of non-recombinant cells as the DsRed marker is located outside the homology arms.

The HDR plasmid donor template is generated through commercial DNA synthesis services.

1. Choose a mammalian expressing vector that accommodates large DNA sequences, such as pUC57-kan (plasmid DNA donor template, Figure 1).

**Figure 1:**
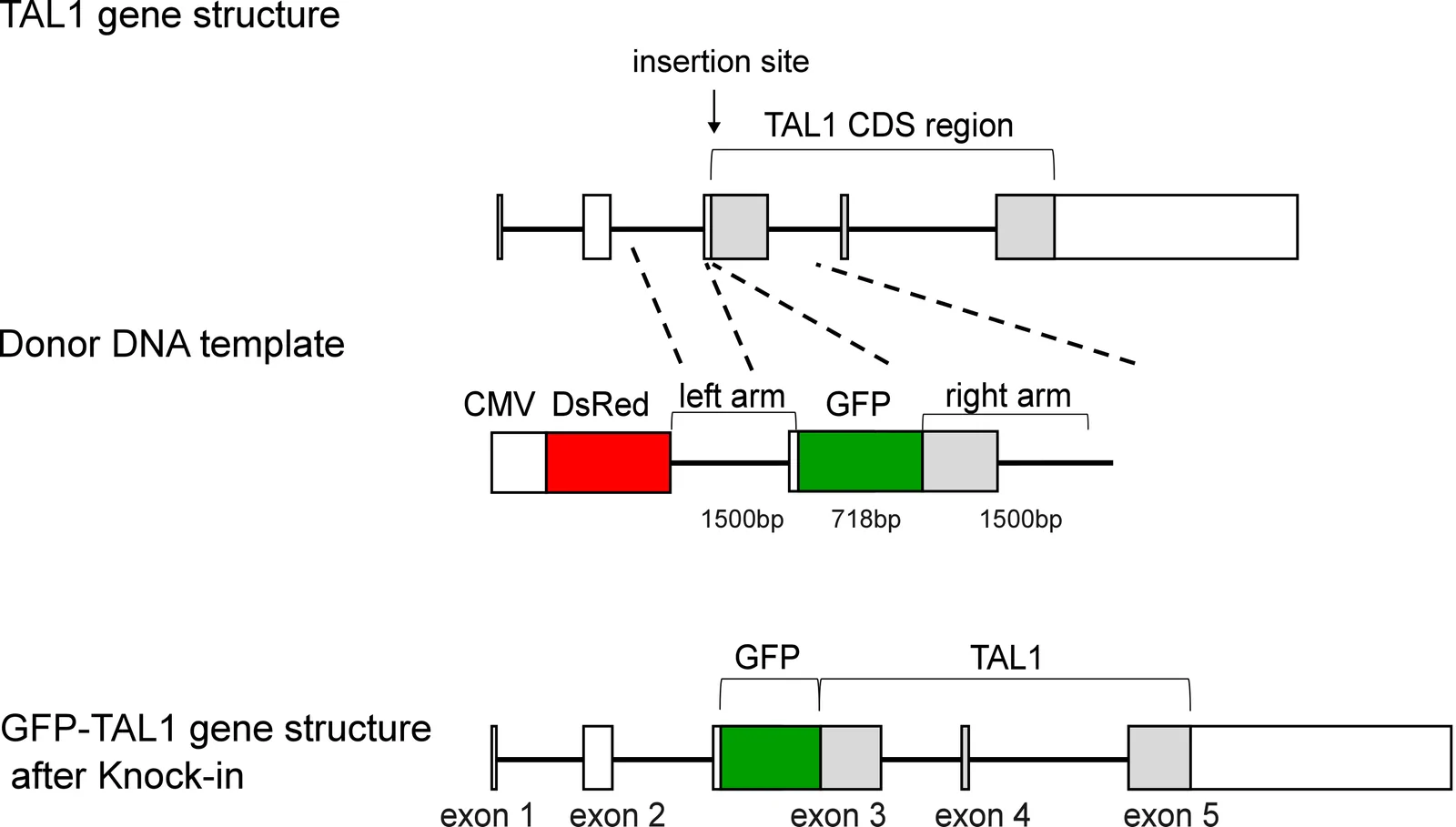
CRISPR-Cas9 HDR strategy to GFP tag the N-terminus of TAL1 TAL1 gene structure showing the insertion site of the GFP fluorescent tag at the N-terminus of TAL1, upstream of exon 3. The TAL1 coding sequence (CDS) is shown in grey. In the donor DNA template, DsRed is placed downstream of the CMV promoter and the GFP is flanked by two homology arms (1500 bp each). Incorporation of blocking mutations in the donor DNA template avoids recognition and re-cutting by Cas9 following successful insertion of the fluorescent tag.The following blocking mutations are incorporated in the right homology arm: ACC>ACA, Threonine; CGG>CGA, Arginine.
2. Utilize a web tool such as Benchling (or CHOPCHOP) to import the DNA sequence of the gene of interest (Figures 2A and 2B).
  a. Bioinformatic tools such as Ensemble or NCBI are embedded into Benchling.
  b. Identify the first coding exon of the gene of interest and highlight the start codon ATG.
    **CRITICAL:** For an N-terminal insertion, the GFP sequence is inserted upstream of the first coding exon and precisely inserted after the start codon, ATG.
  c. On Benchling, select the sequence of the homology arms (sequences homologous to the gene of interest).
    ***Note:*** In the example shown for *TAL1*, the left homology arm is homologous to the 5′ region of exon 3 and the right homology arm is homologous to exon 3 (Figure 1). The left homology arm is placed upstream of the GFP sequence, and the right homology arm is placed downstream of the GFP sequence.
    **CRITICAL:** The length of the homology arms is a determinant factor of the efficiency of HDR.^6^ Larger insertions (>500 bp) require long homology arms (500 bp to 1500 bp), while smaller insertions (<50 bp) can be achieved with shorter homology arms (50–300 bp).
    **CRITICAL:** If repetitive sequences are present, it might be required to increase the length of the homology arms bp to ensure specificity.
    **CRITICAL:** On the plasmid DNA donor template, incorporate blocking mutations in the homology arms (nucleotides highlighted in yellow, Figure 3) to prevent re-cutting by Cas9 following successful incorporation of the fluorescent tag.
    ***Note:*** We recommend a short serine-glycine linker between GFP and the gene of interest (e.g. SGGS) to reduce the chance of interfering with gene function.

**Figure 3:**
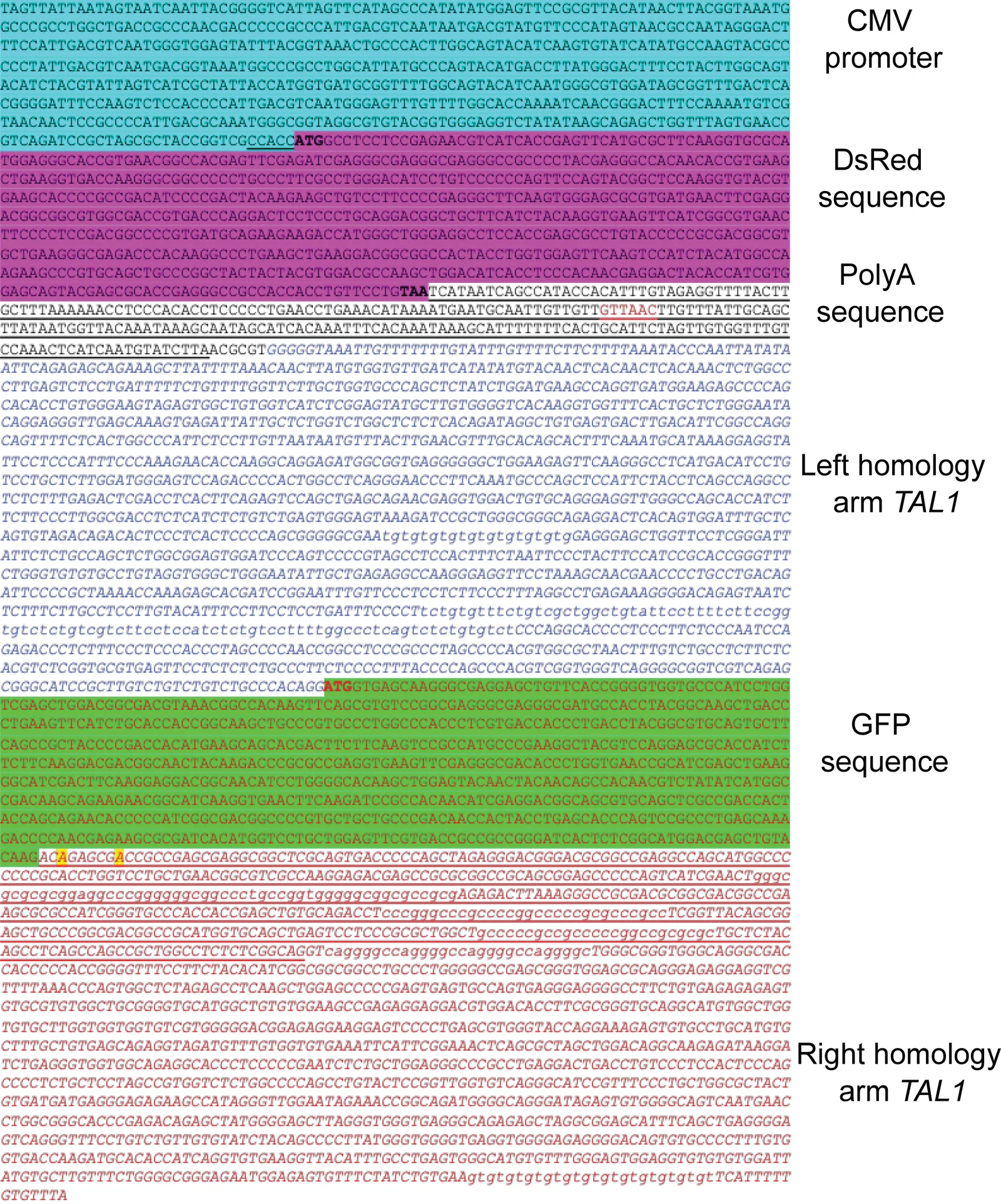
HDR DNA plasmid sequence The HDR template sequence is assembled as follows: CMV promoter- light blue; DsRed- purple; PolyA sequence- black underlined; Left homology arm of *TAL1*- blue; GFP sequence- green; Right homology arm of *TAL1*- red. Nucleotides highlighted in yellow correspond to the incorporated blocking mutations in the homology arm, to prevent re-cutting by Cas9 following incorporation of the GFP fluorophore.

**Figure 2:**
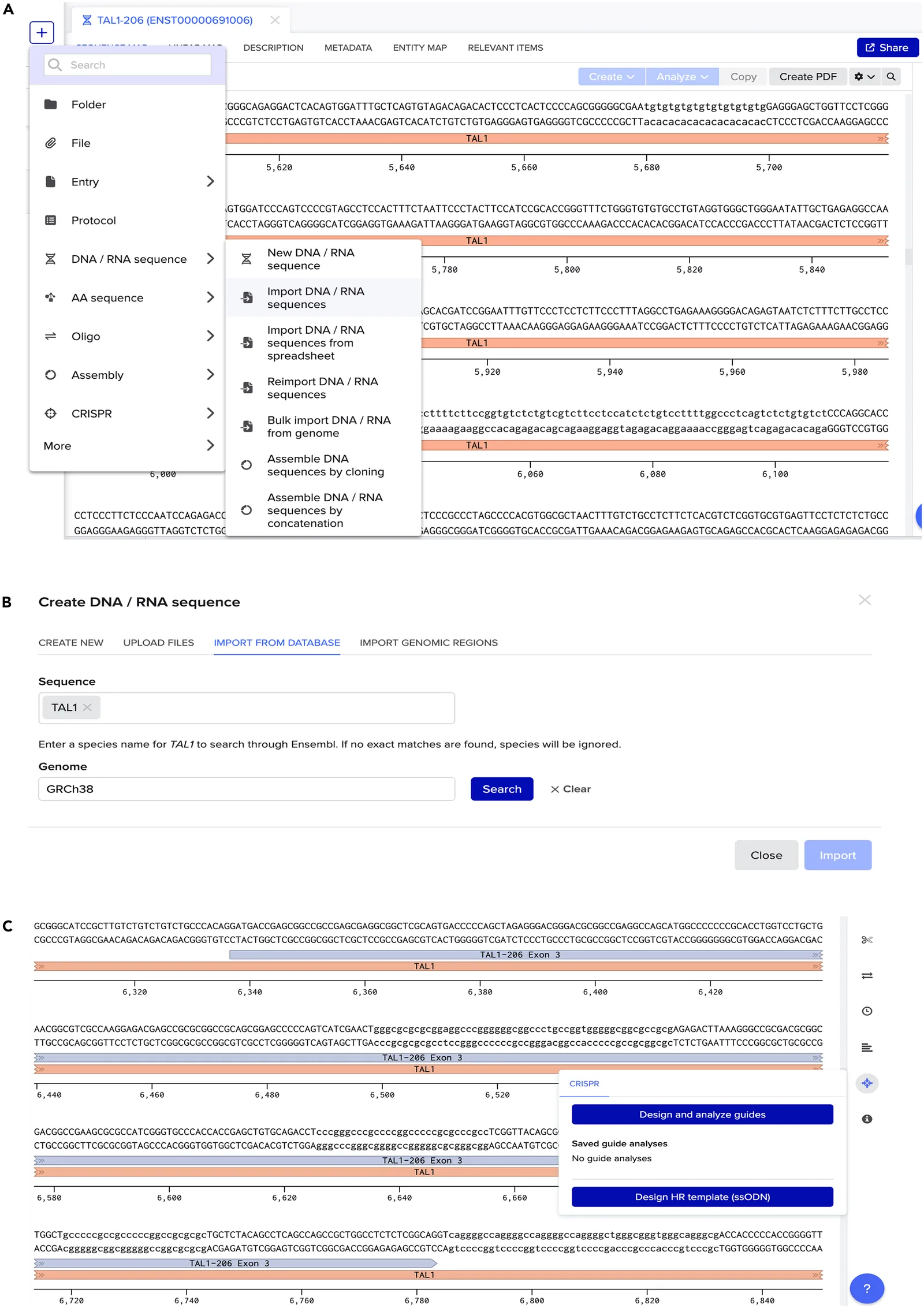
Screenshots of the web tool Benchling, used to assemble the HDR plasmid donor template and to design the sgRNAs (A) and (B) Screenshots showing how to import the TAL1 DNA sequence from a database. (C) Screenshot showing how to design and analyse the sgRNAs.
3. Introduce a DsRed fluorophore sequence downstream of the CMV promoter in the plasmid DNA donor template to monitor cell transfection.
4. Introduce a Poly(A) signal sequence downstream of DsRed.
5. Import the DNA sequence of GFP.
6. Introduce the GFP sequence downstream of the PolyA signal (Figure 3).

**CRITICAL:** If GFP is to be knocked-in to the N-terminus of a gene, remove the stop codon of GFP so that translation of the gene of interest occurs in-frame with GFP. Include a stop codon for C-terminal knock-ins.

7. Generate the DNA plasmid donor template through commercial DNA synthesis services.
8. Design the single guide RNA (sgRNA) sequences using your favored CRISPR design tool.
  a. On Benchling, for instance, use the CRISPR key to design the sgRNA sequences (Figure 2C).

**CRITICAL:** Choose the sgRNA that is the closest to the ATG sequence (or 5′ end of the gene) with the highest “On- Target score” as HDR efficiency is higher when the intended edit is near the cut site.^7^ Below is an example of the sgRNA sequence directed at *TAL1* with the ATG highlighted in red and the PAM highlighted in bold: CTGCCCACAGG**ATG**ACCGAG**CGG**

***Note:*** We suggest testing more than one sgRNA.

***Note:*** Choosing a sgRNA cut site which will be disrupted once a fluorescent tag has been successfully inserted (i.e. knock-in occurs within the sgRNA target site) prevents cutting following successful incorporation of the fluorescent tag sequence.

### Cloning the single guide RNA into the Cas9-expressing vector, followed by bacterial transformation and plasmid amplification

**Timing: 1 week**

The following section describes how to clone the sgRNA sequence into the pX330-U6-Chimeric_BB-CBh-hSpCas9 vector, transform bacterial cells with the construct and screen for positive clones.

9. Order DNA oligonucleotides with the sgRNA sequence (from step 8a) and add the required compatible nucleotides to create sticky ends which will ligate into the linearized mammalian expressing vector.
  ***Note:*** If using pX330-U6-Chimeric_BB-CBh-hSpCas9, leave overhang compatible ends, as follows: 5′-**CACC**[sgRNA oligo sense]-3′ and 5′-**AAAC**[sgRNA oligo antisense]-3’.
  ***Note:*** Add an extra G at the 5′ of the sgRNA if the target sequence does not begin with G. Example of the oligonucleotide sequences are detailed in the key resources Table (KRT).
  a. Resuspend the DNA oligonucleotides in water or 1x TE buffer at the final concentration of 100 μM.
10. Phosphorylate and anneal the DNA oligonucleotides.
  a. Phosphorylate and anneal the oligonucleotides for insertion into the linearized vector, as described in the materials and equipment section.
  b. Dilute phosphorylated and annealed oligonucleotides 1:200.
11. Digest the plasmid and ligate the oligonucleotides.
  a. Digest 5–10 μg of the vector with the appropriate enzyme (px330-U6-Chimeric_BB-CBh-hSpCas9 is digested with BbsI) in a 20 μl reaction volume.
  b. Add 1 μl of CIP (NEB) to prevent re-ligation of the linearized plasmid.
  c. Incubate for 15 min at 37^o^C.
  d. Heat-inactivate by incubating at 65^o^C for 15 min.
  e. Gel purify the digested dephosphorylated vector.
  f. Prepare the ligation reaction into the linearized vector, as described in the materials and equipment section.
12. Transform 2μl of the ligation reaction into 20μl of competent *E.coli* cells using a standard protocol for heat shock transformation.

***Note:*** We recommend the use of chemically competent *Stbl3* cells.

13. After incubating on a bacterial shaker at 37^o^C for 1 h at 200 rpm, take 100μl and spread it onto LB plates containing the appropriate antibiotic.
14. Incubate in an incubator at 37^o^C for 12 to 16 h.
15. Inoculate at least three single clones into 4-5ml LB medium containing the appropriate antibiotic and incubate on a bacterial shaker at 37^o^C, 200 rpm, for 12 to 16 h pre-culturing.
  a. Screen clones by extracting plasmid DNA from 1ml pre-culture using a miniprep kit and sequencing the plasmid DNA using appropriate primers to confirm the insertion.

***Note:*** If using pX330-U6-Chimeric_BB-CBh-hSpCas9, use LKO.1 primer: 5′- GACTATCATATGCTTACCGT-3′ for sequencing

16. Prepare a larger culture from the pre-culture and prepare a midi-prep using an endotoxin-free plasmid purification kit.
17. Determine the plasmid DNA concentration.

### Cell preparation and nucleofection with the CRISPR-Cas9 components

**Timing: 4 h**

In this step, we describe how to deliver the CRISPR-Cas9 components to cells via nucleofection.

18. Thaw plasmids and nucleofection reagents on ice.
19. Prepare a 12-well plate containing 1ml RPMI complete per well to receive the transfected cells.
20. Mix the donor DNA plasmid (from step 7) and pX330-U6-Chimeric_BB-CBh-hSpCas9_sgRNA at equivalent ratios.
21. Harvest 2x10^6^ cells.
  a. Centrifuge at 500 *g* for 5min.
  b. Aspirate supernatant and gently wash cell pellet twice with PBS at 20-25^o^C.

***Note:*** For Jurkat cells,^5^ we recommend splitting and resuspending cells in fresh media the day before editing to ensure optimal growth.

22. Transfect cells via nucleofection.
  a. Resuspend cells gently in 100 μl Ingenio Nucleofector solution (or similar) and add the plasmid mixture.
  b. Transfer resuspended cells into a nucleofection 0.2 mm cuvette and perform nucleofection in an Amaxa device using program D-23.
    ***Note:*** For the Amaxa system, Jurkat cells efficiently transfect using the program D-23.
    **CRITICAL:** Check the appropriate program for the cell line of interest or manually test the programs by setting up individual transfections using 1μg of pMax-GFP per transfection. Evaluate transfection efficiency by FACS analysis.
    ***Note:*** Other high-performance electroporation devices such as the Neon Electroporation System (Invitrogen) are also effective.
  c. Immediately following electroporation, add warm media aspirated from the 12-well plate to the cuvette.
  d. Transfer the electroporated cell suspension to the 12-well plate, avoiding the “foam-like” cell debris.
  e. Place the plate in the incubator at 37^o^C/5%CO_2_.

### Fluorescence-activated cell sorting and expansion of the GFP knockin cells

**Timing: 4 weeks**

In this step, we describe how to select the edited GFP knock-in (KI) cells via FACS and how to expand them.

23. FACS sort GFP KI cells.
  a. After cells recover from nucleofection (around 3 days), centrifuge them and remove the supernatant.
  b. Gently resuspend the cell pellet in FACS buffer (PBS + 2%FBS) and incubate the cell suspension with a viability dye, such as Hoechst 33258 (1:5000 final dilution).
    ***Note:*** Store FACS buffer at 4^o^C for up to 1 week.
  c. Analyze cells on a flow cytometer, selecting the appropriate lasers to detect GFP and DsRed expression.
    ***Note:*** If low transfection efficiency is observed, refer to troubleshooting, problem 1.
    ***Note:*** DsRed positive cells occur when ineffective or off-target recombination have taken place and these cells should be excluded from the sort.
  d. Generate a gate on the viable cells.
  e. Generate a sub-gate on the GFP positive/DsRed negative cell population and sort directly into 96-well plates pre-containing 200 μl of RPMI complete media.
    ***Note:*** If high cell death is observed, refer to troubleshooting, problem 2.
    ***Note:*** Avoid sorting into the outer wells of the plates due to evaporation. Loosely wrap the plates in foil to avoid evaporation.
  f. Incubate cells in the incubator at 37^o^C/5% CO_2_, for a period of 3 to 4 weeks.
24. Upon cell expansion, re-verify GFP expression on a flow cytometer.
25. Extract gDNA from the growing clones using a DNA extraction kit.
26. Perform PCR followed by Sanger sequencing to validate that the fluorescent tag has been successfully inserted without any mutations to the tag or endogenous protein.

***Note:*** If low knock-in efficiency is observed, refer to troubleshooting, problem 3.

27. Expand the clones harboring the GFP tag fused to the oncogene.

### Validation of the GFP knockin cells

**Timing: 2–3 weeks**

This step of the protocol describes how to validate the edited GFP reporter cell line. As an example, we will describe how to target the MYB binding site motif at the non-coding region of the *TAL1* gene.^1^ The MYB binding site is situated 7.5 kb upstream of TAL1 and creates an enhancer that leads to expression of GFP-TAL1 *in cis,* such that its disruption by CRISPR-Cas9 will lead to downregulation of GFP-TAL1 levels.^1^

**Figure 4:**
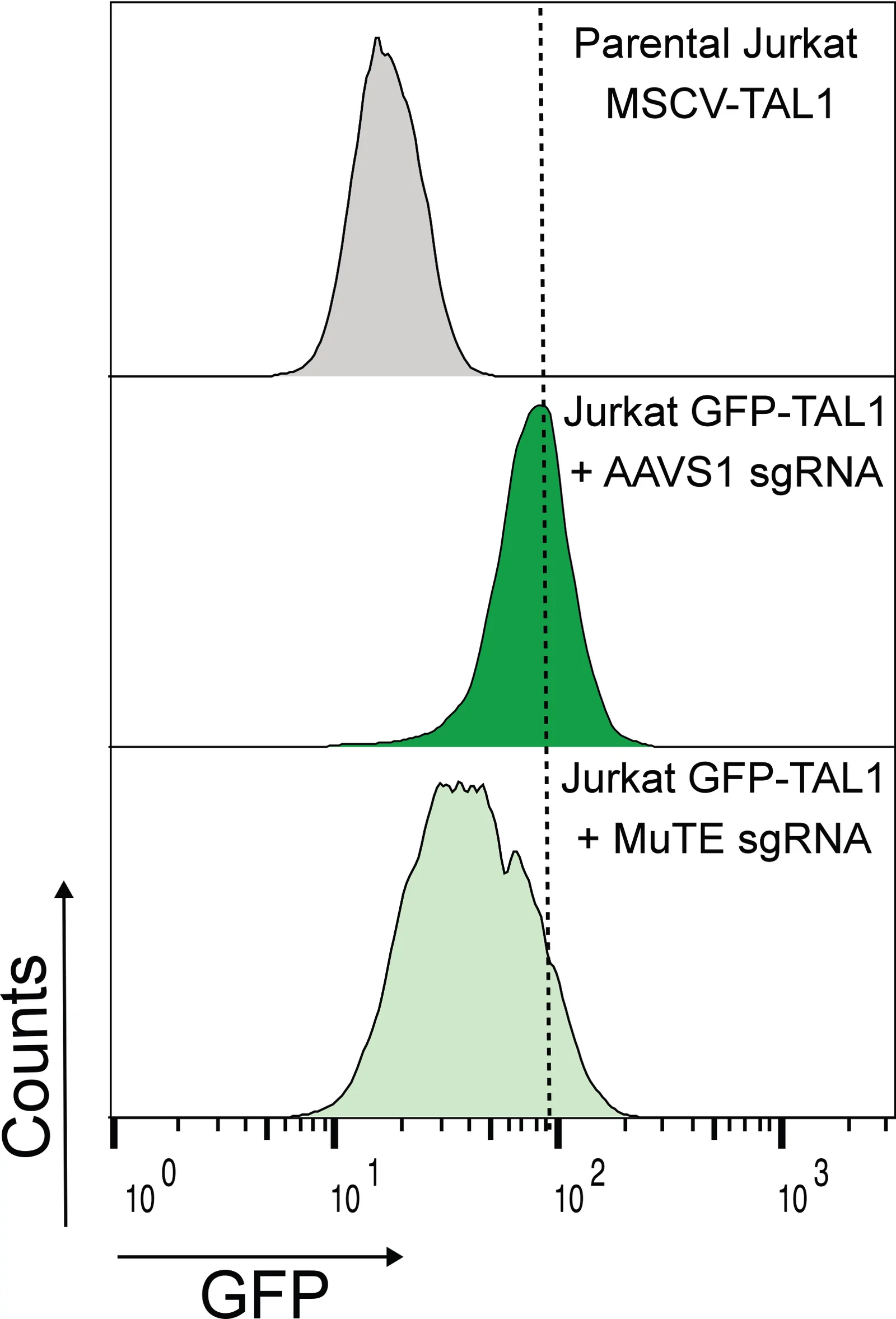
Validation of the reporter Jurkat GFP-TAL1 cell line

28. Abrogate a known regulatory sequence of the fusion GFP-oncogene using the CRISPR-Cas9 RNP strategy.
  a. Using Benchling, select a couple of single guide RNAs targeting a known regulatory sequence of the fusion GFP-oncogene and a single guide RNA targeting a control region (such as the AAVS1 safe harbor locus).
  b. Order the DNA oligonucleotides (crRNA) and tracrRNA labelled with a fluorophore (such as ATTO 550) to monitor transfection efficiency and Cas9.
  c. Resuspend crRNA and tracrRNA in the appropriate buffer at the final concentration of 200 μM.
    **CRITICAL:** Place reagents on ice to avoid degradation/inactivation.
  d. Prepare a 12-well plate containing 1ml RPMI complete per well to receive the transfected cells.
  e. Prepare the RNP complex as follows.
    i. Prepare two mixtures: one containing the sgRNA targeting the locus of interest (sgRNA MYB, see KRT) and the other targeting the safe harbor control locus (sgRNA AAVS1, see KRT).
    ii. Prepare the two-part guide RNA complex by combining crRNA and tracrRNA at 1:1 ratio (e.g., 5μl each).
    iii. Incubate the crRNA:tracrRNA complex at 95^o^C for 5 min. Leave the crRNA:tracrRNA duplexes to cool down to 15-25^o^C.
      ***Note:*** sgRNA complexes can be stored at −20^o^C up to one year.
    iv. Take 3.9 μl of the mixture into a sterile tube and add 5.1 μl of Cas9 protein (10mg/ml) and 6μl of PBS.
    v. Incubate this mixture for 20 min at RT.
  f. Harvest 2x10^6^ cells (per condition).
    i. Centrifuge at 500 *g* for 5 min.
    ii. Aspirate supernatant and gently wash cell pellet twice with PBS at RT.
  g. Perform nucleofection.
    i. Gently resuspend cell pellet in 100 μl nucleofection solution.
    ii. Add 10 μl of the RNP complex (from step 28e).
    iii. Transfer cell suspension into a 0.2 mm cuvette.
    iv. Perform electroporation using Amaxa Nucleofector device (Lonza Bioscience) using the program D-23.
      ***Note:*** If available, use a high-performance electroporation device such as Neon Electroporation System (Invitrogen).
    v. Immediately following electroporation, add warm media -aspirated from the 12- well plate, to the cuvette.
    vi. Transfer the electroporated cell suspension to the 12-well plate, avoiding the “foam-like” cell debris.
  h. Place the plate in the incubator at 37^o^C/5%CO_2_.
  i. Analyse cells 24 h or 48 h after transfection by flow.
    ***Note:*** If high cell death is observed, refer to troubleshooting, problem 2.
    ***Note:*** As this is a transient transfection, analyse cells 48–72 h post-transfection to achieve maximal signal.
    ***Note:*** To evaluate the CRISPR efficiency, PCR amplify the edited locus, perform Sanger sequencing and use bioinformatic tools, such as TIDER,^8^ to evaluate the editing efficiency of the CRISPR-Cas9 experiment.
    ***Note:*** Decreased GFP expression, as compared to control cells (Figure 4), indicates the validity of the cell line. Alternatively, reduction of the GFP levels can be confirmed by qPCR or Western-Blot.

### Perform precise editing of the regulatory region of the GFP-oncogene using CRISPR-Cas9-HDR

**Timing: 1 month**

In this section, we provide a strategy for performing precise editing of the non-coding genome, such as we performed for the GATA3 binding sites that regulate *TAL1* expression in Jurkat cells.^1^ A similar strategy could be used to introduce coding sequence variants at a gene of interest. This strategy uses a short single-stranded DNA oligonucleotide (ssODN) of 100 bp as HDR template carrying the desired sequence.

**Figure 5:**
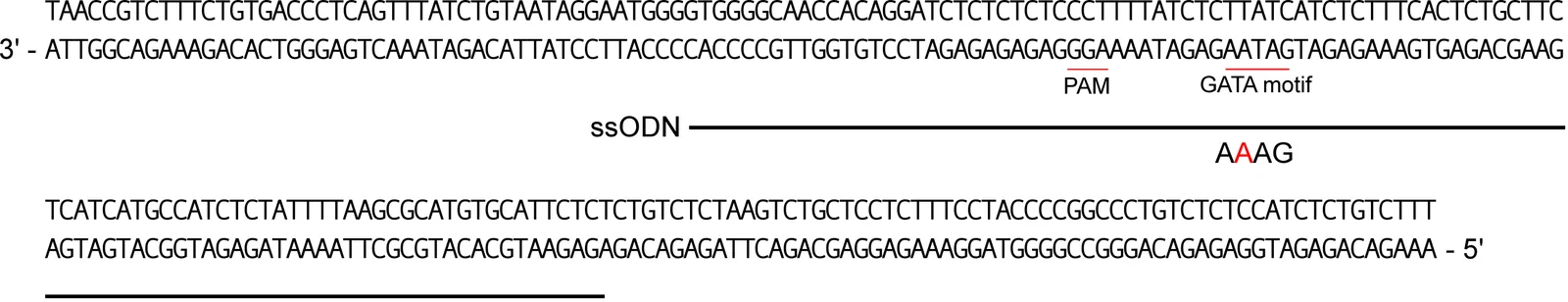
Non-coding regulatory sequence of the oncogene *TAL1* Highlighted in red are the PAM site and the GATA motif 5'-GATA-3'. The ssODN template is 100 bp in length and harbours the modified nucleotide highlighted in red 'A' , disrupting the GATA binding site.
  c. Select a single-stranded oligo donor (ssODN) of approximately 100 bp in length covering the edited site (Figure 5).
  d. Order the crRNAs, tracrRNA and the ssODN.
    **CRITICAL:** Order trans-activating crRNAs (tracrRNA) labelled with a fluorophore that is not the same as the fluorescent reporter gene of the parental cell line, to monitor transfection efficiency.
30. Prepare the sgRNA and ssODN.
  a. Resuspend the following reagents in the appropriate buffer according to the manufacturer’s instructions: single-stranded oligo donor (ssODN), tracrRNA-ATTO550 and custom-designed crRNA targeting the *TAL1* enhancer locus.
  b. Prepare the two-part guide RNA complex.
    i. Combine crRNA and tracrRNA at 1:1 ratio (e.g., 5μl each).
    ii. Incubate at 95^o^C for 5 min.
    iii. Leave the crRNA:tracrRNA duplexes (sgRNA) to cool down to 15-25^o^C.

***Note:*** sgRNA complexes can be stored at −20^o^C up to one year.

29. Design the sgRNA and ssODN.
  a. Import the sequence of the gene locus into Benchling.
  b. Use the CRISPR key to design the sgRNA sequences that are the most efficient and closest to the desired edit site (Figure 5).
    **CRITICAL:** Choose a sgRNA that is closest to the desired edit site to increase HDR efficiency^7^

31. Prepare the RNP complex.
  a. Combine the following components per electroporation per well: sgRNA 3.9μl, 5.1μl Cas9 and 6μl PBS.
  b. Incubate at RT for 20 min, allowing to form a ribonucleoprotein complex (RNP).

***Note:*** RNP complexes can be stored at −80^o^C in single use aliquots.

32. Perform nucleofection on an Amaxa Nucleofector (Lonza Bioscience) using the program D-23.
  a. During the incubation period, harvest 2x10^6^ cells per condition.
    i. Centrifuge at 500 *g* for 5 min.
    ii. Aspirate supernatant and gently wash cell pellet twice with PBS at RT.
    iii. Remove PBS.
  b. Gently resuspend the cell pellet in 100 μL nucleofection solution per condition, with 10 μl of RNP (from step 31) and 3 μl of ssODN at the concentration of 100μM.

***Note:*** If available, use a high-performance electroporation device such as Neon Electroporation System (Invitrogen).

***Note:*** ssODN sequences used for edits in this protocol are listed on Table 1. Modified nucleotides are highlighted in red.

33. Perform single-cell sorting twenty-four hours after nucleofection.
  a. Centrifuge cells at 500 *g* for 5min.
  b. Aspirate supernatant.
  c. Gently resuspend the cell pellet in FACS buffer (PBS + 2%FBS) and incubate the cell suspension with a viability dye, such as Hoechst 33258 (1:5000 final dilution).
  d. Select the top 5% ATTO550 positive cells.
  e. Sort into 96-well plates containing 200 μl RPMI complete per well.

***Note:*** If high cell death is observed before sorting, refer to troubleshooting, problem 2.

***Note:*** If low transfection efficiency is observed, refer to troubleshooting, problem 1.

***Note:*** Use six to eight plates per experimental condition.

34. Expand single-cell clones.
  a. Incubate cells under standard conditions for a two to three-week period (or until a visible cell pellet is seen).
  b. Following single-cell expansion, label each clone and take an aliquot of approximately 50μl.
  c. Expand the remaining cells by transferring them into a 24-well plate.
  d. Isolate gDNA from each clone using QuickExtract DNA Extraction solution (Epicentre).

***Note:*** Elute gDNA in a small volume.

35. Genotype single-cell clones by PCR followed by Sanger sequencing.
  a. Design the PCR strategy, ensuring the edited site is located in the middle of the amplicon.
  b. Optimize the PCR reaction using gDNA from a parental cell line.
  c. PCR amplify the locus of interest using Phusion High-Fidelity DNA polymerase and primers flanking the target edited sites in a 25 μl final reaction, as described in the materials and equipment section.
  d. Take 5 μl of each PCR reaction and run on a 2% agarose gel, 100-110 V (see Figure 6).

**Figure 6:**
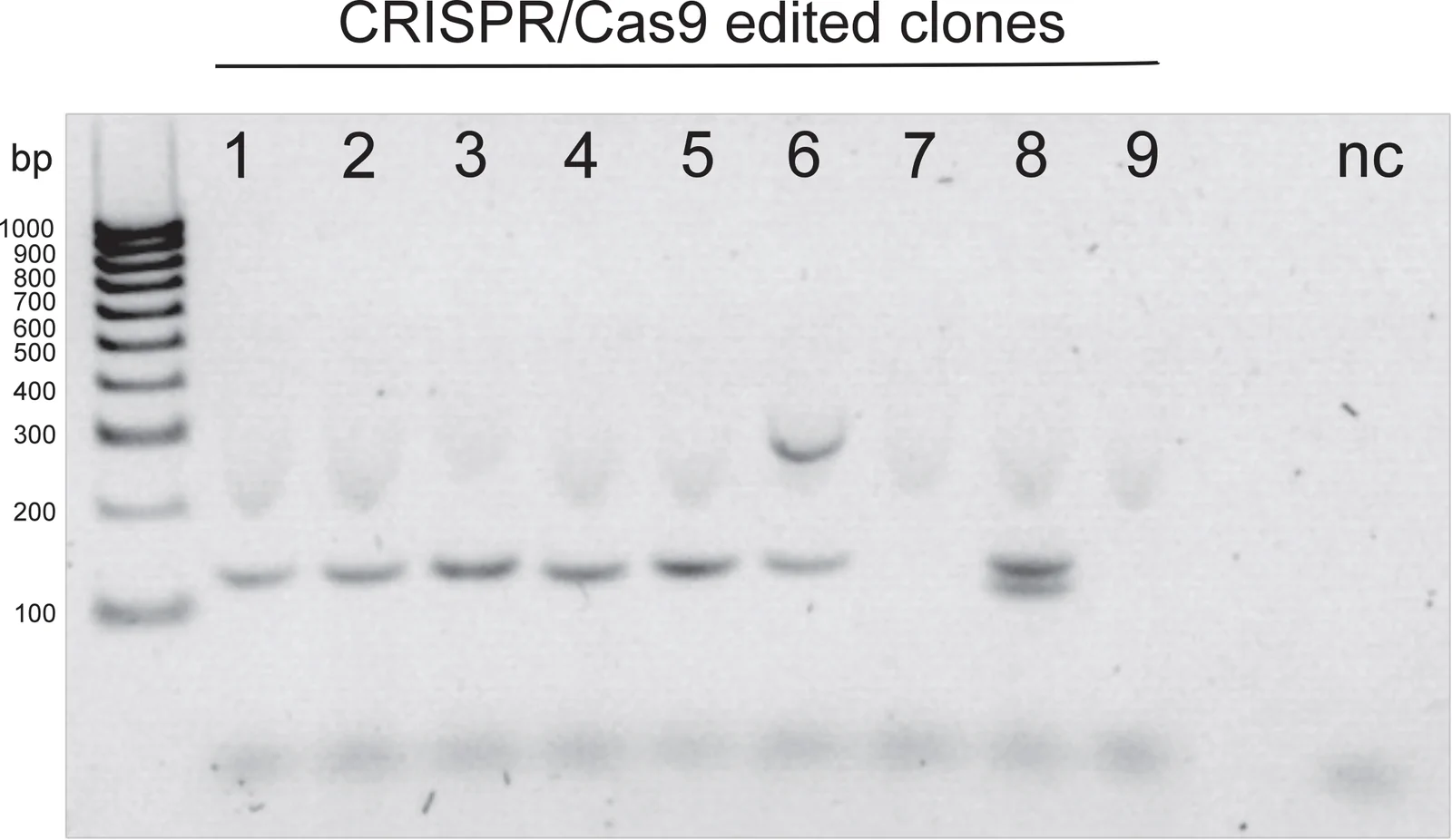
Gel image of the PCR reactions of the amplified gDNA from the CRISPR-Cas9 edited clones GATA Site 3. 2% gel. Amplicon size = 132 bp.
  e. Keep the remaining PCR reaction at 4^o^C for 12 to 16 h or at −20 ^o^C if left for longer.
  f. Make a note of the clones that show a single band.
    ***Note:*** Clones that show more than one band may result from more than one cut (see Figure 6, lanes 6 and 8).
  g. Purify the remaining PCR reaction of the clones that show a single band using a PCR cleanup kit or MinElute.
  h. Confirm the edited loci by Sanger sequencing using the PCR reaction primers.
36. Expand the clones harboring the desired indel by transferring them into a 12-well culture plate.

**Table 1:** sgRNA and ssODN sequences used to generate knock-in cell lines

Target site	sgRNA sequence 5’ – 3′	ssODN template 5’ – 3′
MuTE site	ACAGAAAGACGGTTAGGAAA	GTTGCCCCACCCCATTCCTATTACAGATAAACTGAGG GTCACAGAAAGACGTACCCTACTTCCTGGCAGATGT CTCTGTCACTTTTTCAAATACCATTA
GATA3 S1	ACCCTCAGTTTATCTGTAAT	GTCTTTCTGTGACCCTCAGTTT**T**TCTGTAATAGGAATG GGGTGGGGCAACCACAGGATCTCTCTCTCCCTTTTA TCTCTTATCATCTCTTTCACTCTGCT
CTAT control	ACCCTCAGTTTATCTGTAAT	GTCTTTCTGTGACCCTCAGTTTATCTGTAAAAGGAAT GGGGTGGGGCAACCACAGGATCTCTCTCTCCCTTTT ATCTCTTATCATCTCTTTCACTCTGCT
GATA3 S2	AAGAGATGATAAGAGATAAA	GAGAATGCACATGCGCTTAAAATAGAGATGGCATGA TGAGAAGCAGAGTGAAAGAGATGATAAGAGAAAAAA GGGAGAGAGAGATCCTGTGGTTGCCCCA
GATA3 S3	AAGAGATGATAAGAGATAAA	GAGAATGCACATGCGCTTAAAATAGAGATGGCATGA TGAGAAGCAGAGTGAAAGAGATGAAAAGAGATAAAA GGGAGAGAGAGATCCTGTGGTTGCCCCA
GATA3 S2 & S3	AAGAGATGATAAGAGATAAA	GAGAATGCACATGCGCTTAAAATAGAGATGGCATGA TGAGAAGCAGAGTGAAAGAGATGAAAAGAGAAAAAA GGGAGAGAGAGATCCTGTGGTTGCCCCA

## Expected outcomes

This protocol explains how to use the CRISPR-Cas9 system to tag endogenous proteins in Jurkat T-ALL cells with a GFP tag (Figures 1, 2, and 3). GFP tagging of TAL1 enabled monitoring endogenous gene expression in a genetic screen as well as dissecting the functionality of key regulatory transcription factors.

Clones harboring the GFP knock-in should be validated (Figure 4) before proceeding with following experiments to ensure that tagging of the protein of interest does not significantly alter cellular behavior.

## Limitations

Our protocol does not determine whether the generated knock-ins are heterozygous or homozygous. In our case, this is not relevant as we provided evidence that the knock-ins occur on the enhancer-regulated *TAL1* allele.

In our experiment, the efficiency of knock-ins was 1 in 60–70 single clones examined. Because all sorted cells were ATTO 550 positive (as tracrRNA is labelled with ATTO 550) and not all clones were edited, the presence of ATTO 550 positive cells does not ensure cells have been correctly edited.

Cas9 cuts the genome based on the sgRNA’s recognition of the PAM site. The number of PAM sites at the region of interest may limit the utilization of the CRISPR-Cas9 technology.

Cell lines that are deficient in the DNA repair machinery may be resistant to CRISPR-Cas9, rendering the technique ineffective. For instance, we tried several CRISPR-HDR attempts in the cell line 416 B, however the knock-in success rate was quite low after screening hundreds of clones. The state of ploidy of the cell type of interest may also affect the CRISPR-Cas9 editing efficiency.

## Troubleshooting

### Problem 1

Low transfection efficiency (steps 23 and 33).

### Potential solution 1

Check that cells are growing in log phase and have a viability >90% as too many unhealthy cells will dilute the quantity of RNP complexes delivered to healthy target cells. Ensure the program of nucleofection is the most appropriate for the cell line of interest. Make sure the nucleofection reagents are not expired. Avoid introducing bubbles when resuspending cells in the nucleofection solution or when transferring cells into the electroporation cuvettes.

### Problem 2

High cell-death following transfection (steps 23, 28 and 33).

### Potential solution 1

Make sure the plasmids have been isolated using an endotoxin free kit.

### Potential solution 2

Following electroporation, immediately transfer cells into the plate containing warm media. Avoid leaving cells in the nucleofection buffer before and after electroporation for longer than is necessary.

Avoid use too much HDR DNA template as intracellular ssDNA can be recognized as a ‘foreign DNA species’.

### Potential solution 3

Optimize the electroporation settings for the cell line of interest. Use a pMAX-GFP plasmid as a control and select the program that results in the highest percentage of GFP positive cells with the lowest level of cell death.

### Problem 3

Low Knockin efficiency (step 26).

### Potential solution 1

Use an alternative sgRNA around the desired site of the knock-in.

### Potential solution 2

Increase the number of single sorted cells.

### Problem 4

Low number of heterozygous knock-in clones (step 35).

### Potential solution 1

Use a double ssODN strategy, where two donor templates are introduced simultaneously: one harbouring the mutation of interest and the other containing the wild-type sequence. This increases the likelihood of generating ‘clean’ heterozygous knock-ins.

## Resource availability

### Lead contact

Further information and requests for resources and reagents should be directed to and will be fulfilled by the lead contact, Marc R. Mansour (m.mansour@ucl.ac.uk).

### Technical contact

Technical questions on executing this protocol should be directed to and will be answered by the technical contact, Joana R. Costa (joana.rodrigues-simoes-da-costa@lsbu.ac.uk).

### Materials availability

All plasmids and cell lines generated in this study are available upon request from the lead contact with a completed materials transfer agreement (MTA).

### Data and code availability

• No original code is generated in this study.
• Any additional information required will be available from the lead contact upon request.

## Acknowledgments

The authors would like to thank all past and present members of the Mansour Lab for the assistance, insightful advice, and discussion that helped make this work possible. This work was funded by a 10.13039/501100000289CRUK Programme Foundation Award (award DRCPFA\100012) and the 10.13039/501100000402Kay Kendall Leukaemia Fund (J.R.C.). Y.L. was funded by a John Goldman Award from Leukaemia UK. S.A. was funded by Hardy Keinan Fellowship. M.R.M. is funded through a Great Ormond Street Children’s Charity Professorship. T.R.S. was funded through a Children with Cancer grant.

## Author contributions

J.R.C. and M.R.M. conceptualized the study. J.R.C., S.A., and Z.L. designed and created knockin cell lines. T.R.-D., M.E.K., and S.H. helped screening the edited lines. G.M. and Y.G. performed cell sorting experiments. J.R.C. and Y.L. prepared the figures. J.R.C., Y.L., and M.R.M. contributed to critical discussions and generated the first draft of the manuscript. M.R.M. supervised the work. All authors contributed to critical discussions and approved the final manuscript.

## Declaration of interests

Z.L. is currently employed by Elsevier as a scientific editor at *Cancer Cell*. His contributions to this study were performed prior to joining Elsevier, during his postdoctoral training at Dana-Farber Cancer Institute.
